# In-silico mining and characterization of MYB family genes in wilt-resistant hybrid guava (*Psidium guajava* × *Psidium molle*)

**DOI:** 10.1186/s43141-023-00528-3

**Published:** 2023-06-30

**Authors:** Israr Ahmad, Sumit K. Soni, Muthukumar M., Devendra Pandey

**Affiliations:** Division of Crop Improvement and Biotechnology, ICAR-Central Institute for Subtropical Horticulture, Rehmankhera, P.O. Kakori, Lucknow, Uttar Pradesh 226101 India

**Keywords:** In silico mining, MYB, *Psidium guajava*, RT-PCR

## Abstract

**Background:**

The MYB family is one of the most significant groups of transcription factors in plants. However, several MYBs have been linked to secondary metabolism and are important for determining the color of fruit’s peel and pulp. Despite being a substantial fruit crop in tropical and subtropical areas of the world, wilt-resistant hybrid guava (*Psidium guajava* × *Psidium molle*; PGPM) has not yet been the subject of a thorough examination. This study’s goal was to assess the expression of MYB in guava fruit pulp, roots, and seeds to predict its function by in silico analysis of the guava root transcriptome data.

**Results:**

In the current study, we have mined the MYBs family of MYB genes from the transcriptome of the PGPM guava root. We have mined 15 distinct MYB transcription factor genes/transcripts viz *MYB3*,* MYB4*,* MYB23*,* MYB86*,* MYB90*,* MYB308*,* MYB5*,* MYB82*,* MYB114*,* MYB6*,* MYB305*,* MYB44*,* MYB51*,* MYB46*, and *MYB330*. From the analyses, it was found that R2-MYB and R3-MYB domains are conserved in all known guava MYB proteins. The expression of six different MYB TFs was examined using semi-quantitative RT-PCR in “Shweta” pulp (white colour pulp), “Lalit” pulp (red color pulp), “Lalit” root, and “Lalit” seed.

**Conclusion:**

There were 15 MYB family members observed in guava. They were unequally distributed across the chromosomes, most likely as a result of gene duplication. Additionally, the expression patterns of the particular MYBs showed that MYB may be involved in the control of wilt, fruit ripening, seed development, and root development. Our results allow for a more thorough functional characterization of the guava MYB family genes and open the door to additional research into one essential MYB transcription factor family of genes and its involvement in the growth and ripening of guava fruit.

**Supplementary Information:**

The online version contains supplementary material available at 10.1186/s43141-023-00528-3.

## Background

Multigene families govern the growth and development of plants. A DNA-binding domain, a nuclear localization signal, a transcription activation domain, and an oligomerization site are the four primary domains that transcription factors typically contain and that play a significant role in the regulation of gene transcription [1]. Through the activation or suppression of the transcriptional process, these four domains cooperate to regulate a wide range of aspects of plant development and growth [2]. Analysis of each transcription factor’s distinct roles is complicated by the fact that multigene families frequently encode transcription factors [1]. The MYB transcription factors of higher plants are more widely distributed across the genome than those of fungi and mammals [3].

MYB transcription factors are made up of two unique regions: an N-terminal conserved MYB DNA binding domain and a C-terminal variable modulator region that regulates the activities of proteins. Plants have a high degree of conservation of the MYB domain, at N-terminus and proteins typically contain one to four repeats viz R1, R2, R3, and R4. Each repetition contains 50–53 amino acids that code for three α-helices, from which the second and third α-helices form the HTH (helix-turn-helix) structure [4]. The third α-helix interacts with the major groove of DNA and forms the transcription factor DNA recognition site [5]. A set of highly conserved tryptophan (W) residues found in the MYB domain is involved in sequence-specific DNA binding [6]. Contrarily, the C-terminal, promoter domain of various MYB proteins is extremely varied, contributing to the wide range of regulatory tasks played by the MYB gene family [7–9]. Higher plants often include R2R3-MYB domain proteins as their predominant form [10]. The regulation of primary and secondary metabolism, the management of the cell cycle, and the response to abiotic and biotic stressors are just a few of the phases of plant growth and development where the plant MYB transcription factors have been shown to play a role [11, 12].

The guava (*Psidium guajava* L.; PG) is a significant fruit crop in tropical and subtropical regions of the world. Guava has 2n = 22 chromosomes and a genomic size of over 450 MB. It belongs to the family Myrtaceae, which comprises about 150 species [13]. Guava is mostly produced in India, Mexico, Pakistan, Taiwan, Thailand, Colombia, and Indonesia, with small-scale plantations also operating in Malaysia, Australia, and South Africa [14]. There are approximately about 400 guava varieties produced all over the world, each with a unique fruit pulp and peel colour. When a fruit reaches maturity, the peel changes from green to yellow or red, and the fruit pulp can range from white to deep pink. This trait varies between cultivars and depends on the climate [15].

Consumers typically choose visually appealing colorful fruits because they have better nutritional characteristics and attractiveness. Genes involved in secondary metabolic pathways, particularly phenylalanine ammonia-lyase (PAL), anthocyanidin synthase (ANS), dihydro-flavonol 4-reductase (DFR), chalcone synthase (CHS), flavanol synthase/flavanone 3-hydroxylase (F3H), UDP-glucose: flavonoid 3-O-glucosyltransferase (UFGT), MYB transcription factors, basic helix-loop-helix (bHLH), tryptophan-aspartic acid repeat set c. regulate the color of fruits and vegetables [16–21].

Although *Psidium guajava* L (PG) is a lucrative economic crop it suffers huge losses due to wilt susceptibility. The specific etiology of wilt is unknown, but it has been linked to the pathogens *Fusarium* *oxysporum*, *Fusarium* *solani*, *Rhizoctonia bataticola*, *Macrophomina phaeseoli*, *Gliocladium* *roseum*, and *Cephalosporium sp.* etc. To combat wilt, the ICAR-Central Institute for Subtropical Horticulture in Lucknow has developed an interspecific hybrid rootstock of guava hybrid *Psidium guajava* × *Psidium molle* (PGPM). The rootstock has been demonstrated to be wilt-resistant and grafted successfully with commercial guava varieties [22, 23]. In light of the foregoing, it was hypothesized that the MYB also play a crucial role in determining the peel and pulp colour of guava fruit in PG as well as wilt resistance in PGPM. The first step for alteration via orderly breeding or genome editing is the identification of MYB candidate genes and the controlled pathways [24, 25]. The scarcity of genomic data for guava presents a significant challenge for genetic study. However, the development of genomic and transcriptomic analytical resources and tools is being aided by NGS techniques and bioinformatics pipelines. No attempt has been made to describe MYB genes implicated in guava development, even though physiological studies have been conducted. The objective of this study was to evaluate MYB expression in guava fruit pulp, root, and seed to determine its function by in silico analysis of the root transcriptome data of guava.

## Materials and method

### Transcriptome sequencing and in silico analysis

On the Illumina platform, paired-end sequencing was used to analyze the transcriptome sequence of the PGPM. The libraries were created using the Illumina TruSeq Stranded Total RNA Library Preparation Kit following the manufacturer's instructions using input total RNA of less than 1 g. Using Trinity software at default settings, high-quality reads were achieved. Trinity software was used with the default parameters to produce high-quality reads. The CD-HIT software was used to further process the transcripts for the prediction of Unigenes. With the help of blast analysis, more than 35,000 CDS were located with precise gene annotations. Nucleotide sequences were analyzed using nucleotide BLAST against mango genome data in the National Center for Biotechnology Information to determine the chromosomal placement and location of MYB CDS (NCBI). Using the online sequence manipulation suite (https://www.bioinformatics.org/sms2/), the nucleotide CDS sequence was converted into amino acid sequences. The MOTIF Search tool (https://www.genome.jp/tools/motif/) was used to analyze the motif search results. Using the MUSCLE tool (https://www.ebi.ac.uk/Tools/msa/muscle/), multiple amino acid sequence alignments of MYB proteins were carried out using CDS of transcriptome data. Weblogo (https://weblogo.berkeley.edu) tool was used to prepare the logo sequences for the R2-R3-MYB domains of MYB proteins. The gene expression analysis of mine MYB family genes was carried out using commonly occurring CDS (based on common NR blast hit accession) taking as control with hybrid *Psidium guajava* × *Psidium molle* hybrid (wilt-resistant) as treated. The Gene Ontology (GO) and Subcellular localization analysis of the mine MYB family genes was performed using the online web-server BUSCA (Bologna Unified Subcellular Component Annotator) (https://busca.biocomp.unibo.it/). The phylogenetic position of mine MYB family genes with respect to the reference MYB gene of *Arabidopsis thaliana* (AAD46772) was performed using MEGA version 5.2 software. The protein sequence of mine MYB family genes was aligned through by inbuilt MUSCLES alignment tool of MEGA version 5.2 software. The evolutionary history was inferred by using the Maximum Likelihood method based on the Poisson correction model with bootstrap replications of 500. Initial tree(s) for the heuristic search were obtained automatically by applying Neighbor-Join and BioNJ algorithms to a matrix of pairwise distances estimated using a JTT model, and then selecting the topology with superior log likelihood value [26].

### Semi-quantitative RT-PCR analysis

Lalit (pulp, seed, and root) and Shweta (pulp) samples of frozen guava were pulverized to a fine powder with liquid nitrogen using a mortar and pestle. Total RNA was isolated using the Spectrum TM plant total RNA kit (Sigma, USA) following the manufacturer's instructions. NanoDrop was used to monitor the purity and quantity of total RNA. The Maxima first strand cDNA synthesis kit for RT-qPCR (Genetix) was used to reverse transcribe 2 g of total RNA from each sample using OligodT and random primers under the manufacturer guidelines. A 1:5 dilution of the synthesized cDNA in nuclease-free water was performed before qRT-PCR analysis. The following parameters were employed to develop primer (Table 1) using IDT PrimerQuest software and the CDS acquired from fruit transcriptome data: OligoAnalyzer was used to check for the presence of stable hairpins and dimers. The ideal length was 25 base pairs, the GC content was 50–55%, the melting point was 57 °C, and the amplicon length range was 100–200 base pairs. For in silico confirmation of each gene’s specificity, the produced primer pair was then aligned to all guava CDS. One hundred nanograms of cDNA, 0.5 µm conc. of each primer, 2.5 mM dNTPs, and 1 unit of Taq DNA polymerase were mixed with 1 × PCR buffer to perform PCR amplification in a total volume of 10 µl. The reaction was run through 35 cycles in a Bio-Rad thermal cycler, starting with an initial denaturation at 94 ℃ for 30 s, followed by 57 °C for 30 s, 72 ℃ for 30 s, and a final extension at 72 °C for 5 min. PCR-amplified products were visualized via a trans-illuminator after being resolved on a 2.0% agarose gel made with 1 × TBE buffer and 0.5 µg of ethidium bromide.

**Table 1:** Primers used in semi-quantitative RT-PCR analysis

Gene	Forward primer (5′-3′)	Reverse primer (5′-3′)
*MYB3*	GACCATCATTCAACTCCATA	TCTTCCTGATGTGGGTGTTCC
*MYB4*	CTTGTGGTCTCCAGAGGAAG	CAGGCCTCAAGTAATTGATCCA
*MYB23*	AACCAGACATCAAGAGAGGT	GTGGTGTTCCAGTAGTTCTTGA
*MYB86*	TTGAGGCCTGATTTGAAGAG	AAGAGTTCCACAGATTCTTG
*MYB90*	CAAGGGAGCATGGACGGCAG	CGGAGATGTTCCCTCTCTTA
*MYB308*	AACTCCGGACGAGGACGATC	TGGGTGTTCCAGTAGTTCTTG

## Result and discussion

**Fig. 1:**
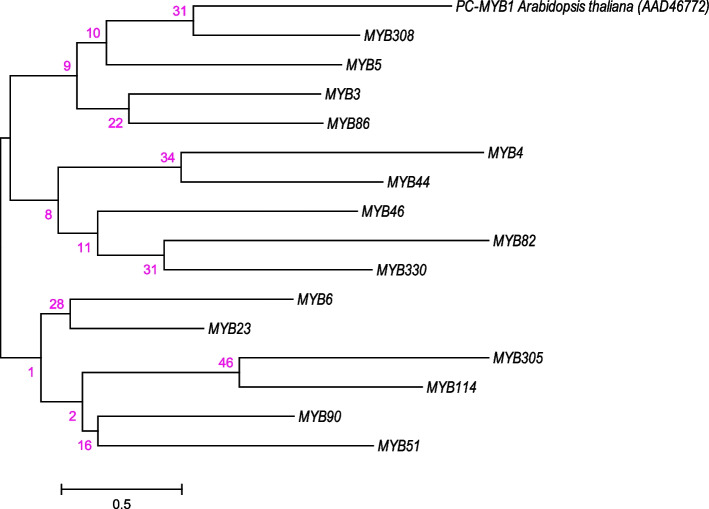
Molecular phylogenetic analysis by maximum likelihood method. The tree with the highest log likelihood (-2952.9718) is shown. The percentage of trees in which the associated taxa clustered together is shown next to the branches. The tree is drawn to scale, with branch lengths measured in the number of substitutions per site. The analysis involved 16 amino acid sequences. All positions containing gaps and missing data were eliminated. There were a total of 65 positions in the final dataset

In this study, 2.64 GB of data was generated from root transcriptome analysis of the PGPM. Trinity software was used to do a de novo assembly of high-quality reads, identifying 170,027 transcripts with a maximum length of 962 bp. The top-hit species distribution showed that the species *Eucalyptus grandis* received the bulk of hits. We have mined 15 different MYB transcription factors genes/transcripts (*MYB3*, *MYB4*, *MYB5*, *MYB6*, *MYB23*, *MYB44*, *MYB46*, *MYB51*, *MYB86*, *MYB82*, *MYB90*, *MYB114*, *MYB305*, *MYB308*, and *MYB330*) in the root transcriptome data of guava. Further, the phylogenetic position of mine MYB family genes with respect to the reference MYB gene of *Arabidopsis thaliana* (AAD46772) is depicted in Fig. 1. Phylogenetic analysis grouped the mine MYB family genes into four clusters.

**Fig. 2:**
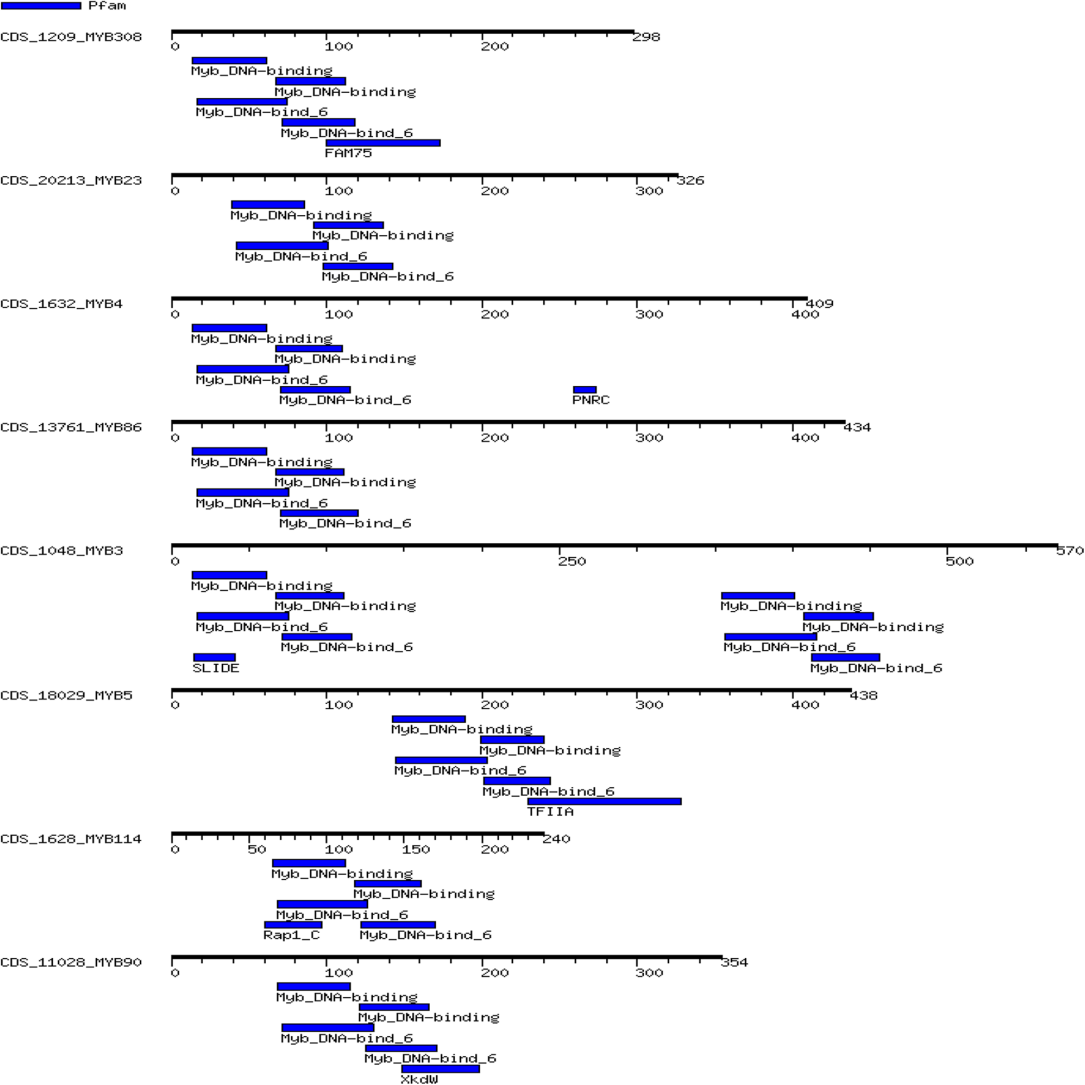
Distribution of MYB protein domains and motifs in different CDS of guava

**Fig. 3:**
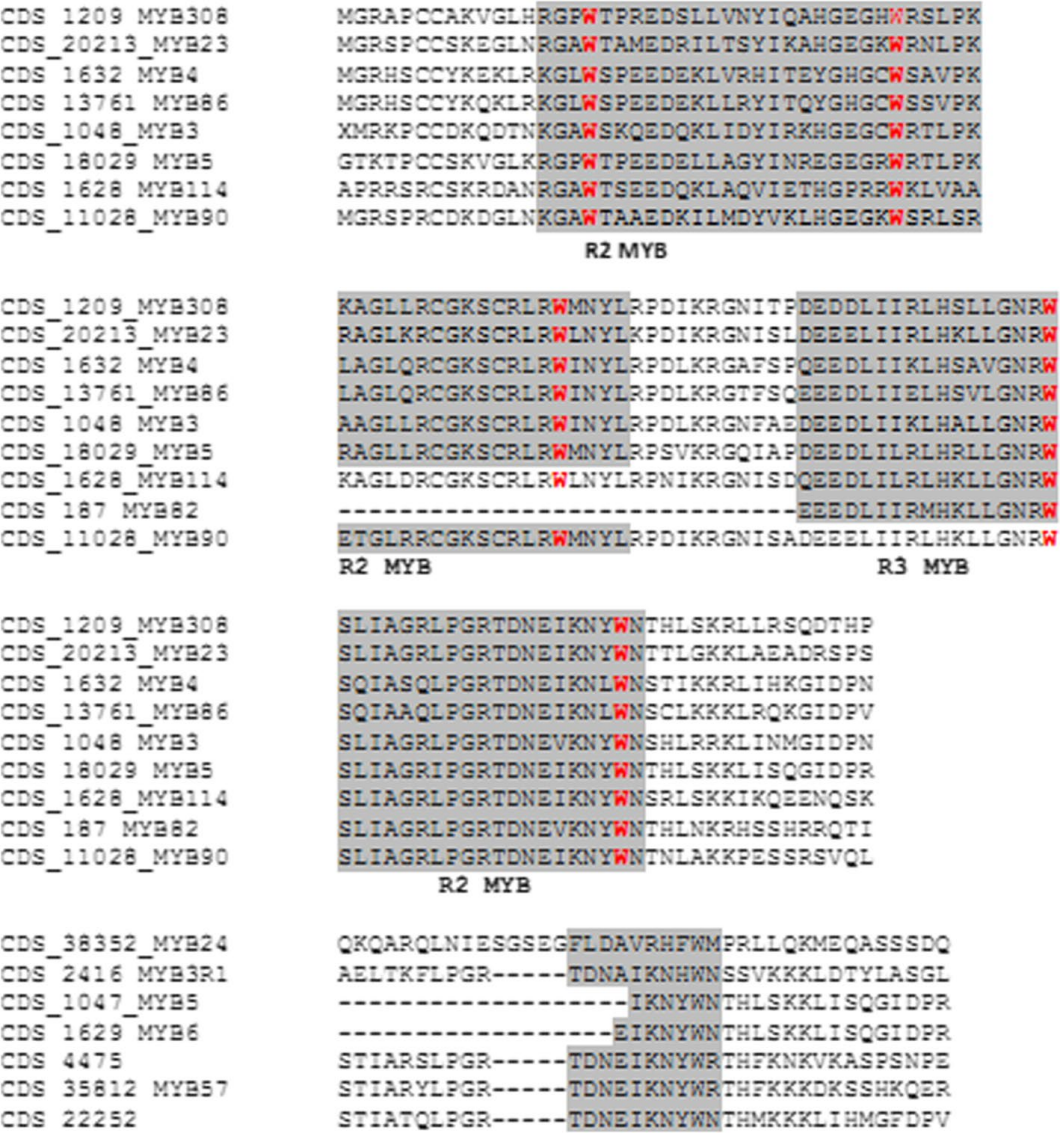
Multiple sequence alignments of MYB proteins. **a** R_2_MYB. **b** R_3_MYB of guava

**Fig. 4:**
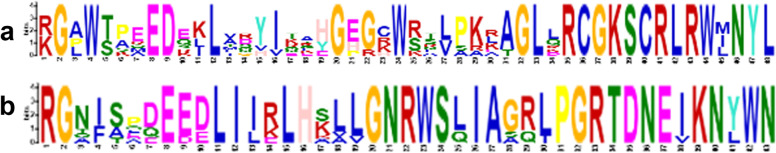
Logo sequences for **a** R_2_MYB, **b** R_3_MYB domain of guava MYB proteins prepared from Weblogo

We used a bioinformatics technique to study domain analysis in coding sequences, which reveals that all mined MYB genes possess the conserved R2-MYB and R3-MYB domains and were found to be unevenly distributed throughout guava chromosomes (Fig. 2; Table 2). WebLogo was used to build sequence logos that could be used to show conservation at specific points. The conserved amino acids shared by MYB domains were strikingly similar, as seen in Figs. 3 and 4. According to these findings, the R2 and R3 repeats of the guava MYBs proteins contain a large number of conserved amino acids, including the distinctive Trp (W). The R2 repeat contains three conserved Trp (W) residues. The R3 repeat only conserved the second and third Trp (W), while the first Trp (W) was frequently replaced with phenylalanine (F) or isoleucine (I) (Figs. 3 and 4). It is possible that substitution at the first Trp (W) residue causes the recognition of new target genes and/or results in a reduction in the DNA-binding activity against target genes. The C-terminal domain is more mutable, whereas the N-terminal domain is more conserved [6, 27]. Gene Ontology (GO) and Subcellular localization analysis of the identified MYB family genes revealed that *MYB5*, *MYB3*, *MYB1*, *MYB308*, *MYB51*, *MYB86*, *MYB4*, *MYB46*, *MYB23*, *MYB330*, *MYB114*, *MYB305*, *MYB44* were localized in the nucleus. Whereas the subcellular localization of *MYB90* and *MYB5* accounted for chloroplast and endomembrane systems respectively (Table 3 and supplementary data).

**Table 2:** Chromosomal location and nucleotide positioning of different CDS sequences having R2–R3 MYB domain

CDS_MYB	Chromosome (+/−)	Chromosomal location
CDS_187_MYB82	6 (+)	11203042 to 11203088 11203200 to 11203555
CDS_1047_MYB5	10 (+)	29397091 to 29397666
CDS_18029_MYB5	5 (+)	12762141 to 12762789 12763182 to 12763853
CDS_1048_MYB3	7(−)	30879921 to 30880054 30879617 to 30879750 30878781 to 30879528
CDS_1145_MYB1R1	5 (−)	42430294 to 42430731
CDS_1209_MYB308	9 (+)	1960510 to 1960643 1960848 to 1961612
CDS_1628_MYB114	6 (−)	5437874 to 5438160 5437180 to 5437492
CDS_1629_MYB6	1(+)	13565271 to 13565861
CDS_1632_MYB4	5 (+)	16370726 to 16370859 16370961 to 16371093 16371186 to 16372155
CDS_2416_MYB3R1	6 (+)	32952467 to 32954264 32954612 to 32954946 32955435 to 32955672 32952238 to 32952369 32956171 to 32956297
CDS_4475_MYB305	4 (−)	27474181 to 27474727
CDS_4529_MYB44	3 (+)	48186292 to 48186660
CDS_11028_MYB90	6 (+)	16927844 to 16928140 16929089 to 16929730
CDS_12868_MYB51	1 (+)	38436377 to 38437214
CDS_13761_MYB86	2 (+)	17474766 to 17475163
CDS_18309_MYB46	5 (+)	13524439 to 13524804
CDS_20213_MYB23	6 (−)	5306240 to 5306885 5307239 to 5307444 5306968 to 5307107
CDS_21499_MYB330	6 (−)	6631103 to 6631645
CDS_22252 _MYB330	3 (+)	45943958 to 45944765
CDS_35812	8 (+)	11169084 to 11169587
CDS_38352	5 (−)	6987291 to 6987668
CDS_32049_MYBAPL	3 (−)	23666873 to 23667736

**Table 3:** Gene ontology (GO) and Subcellular localization analysis of the identified MYB family genes

S.N	Accession	GO ids	GO terms	Score	Alternative localization
1	*MYB5*	GO:0005634	C: nucleus	1	
2	*MYB3*	GO:0005634	C: nucleus	1	
3	*MYB90*	GO:0009507	C: chloroplast	1	
4	*MYB1*	GO:0005634	C: nucleus	1	
5	*MYB308*	GO:0005634	C: nucleus	1	
6	*MYB51*	GO:0005634	C: nucleus	1	
7	*MYB86*	GO:0005634	C: nucleus	1	
8	*MYB4*	GO:0005634	C: nucleus	1	
9	*MYB5*	GO:0012505	C:endomembrane system	0.81	GO:0031090-C:organelle membrane (score = 0.71)
10	*MYB46*	GO:0005615	C:extracellular space	0.62	GO:0005634-C: nucleus (score = 0.38)
11	*MYB23*	GO:0005634	C: nucleus	1	
12	*MYB330*	GO:0005634	C: nucleus	1	
13	*MYB114*	GO:0005634	C: nucleus	1	
14	*MYB305*	GO:0005634	C: nucleus	1	
15	*MYB44*	GO:0005615	C: extracellular space	0.62	GO:0005634-C: nucleus (score = 0.38)

**Fig. 5:**
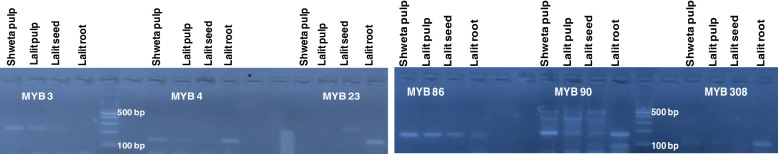
Semi-quantitative RT-PCR analysis of different MYB in root, pulp, and seed of guava

**Fig. 6:**
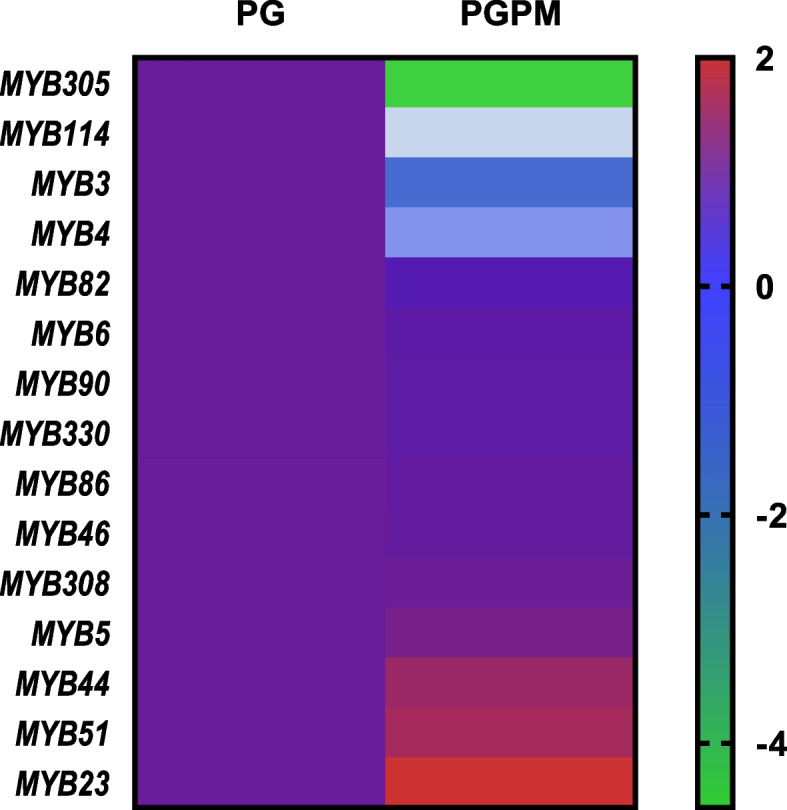
Heatmap of upregulated and downregulated 15 mine MYB gene. Scaling is the base mean value, i.e., dark red colour represents upregulated genes whereas green colour represents downregulated genes. MYB family genes of PG (*Psidium guajava*) taking as control while PGPM (hybrid *Psidium guajava* × *Psidium molle*) is treated

The expression of *MYB3*, *MYB4*, *MYB23*, *MYB86*, *MYB90*, and *MYB308* was also evaluated in the Lalit fruit pulp (red pulp), seed, root, and Shweta pulp (white pulp) (Fig. 5). *MYB23* and *MYB308* were only expressed in the root tissue, indicating that they play a crucial role in the development of roots. While pulp and root tissue showed *MYB4* expression (Fig. 4). *MYB86* and *MYB90* expression was identified in all tissues, including the root, seed, and pulp, indicating that they play a significant role in the development of fruits and roots (Fig. 4). *MYB3* was only expressed in the fruit pulp, highlighting its crucial function in fruit ripening (Fig. 4). The heatmap analysis also accounted the exclusive significant higher expression of *MYB23* gene in wilt-resistant PGPM as compared with wilt susceptible *Psidium guajava* (Fig. 6). This result indicates that higher expression of *MYB23* (only expressed in root) gene might play a crucial role to confer the wilt resistance in PGPM.

Gene functions can be better understood by understanding the patterns of gene expression. Recent research has revealed that the pear’s skin, bud, and fruit express MYB genes [28, 29]. Through their conserved N-terminal domains and their C-terminal domains, which are particular to transcriptional activation or repression, conserved MYB TFs can take part in intricate physiological processes. For instance, *MYB3*, *MYB86*, and *MYB90* were involved in fruit ripening and *MYB4*, *MYB90*, and *MYB308* in root development (Fig. 4). Previous studies on apples, grapes, and strawberries by various researchers provided support for this finding [30–33]. They discovered that MYB-bHLH-WD40 complexes controlled the transcription of the genes responsible for producing structural anthocyanins. In a variety of plants, including *Arabidopsis thaliana* [6], *Malus domestica* [34], *Solanum tuberosum* [35], *Solanum lycopersicum* [36], *Gossypium raimondii* [37], etc., the roles of MYB proteins have been thoroughly studied. The MYB gene in *Arabidopsis thaliana* controls the production of flavonoids in most tissues [38]. This pathway appears to be extremely conserved because orthologs of the *AtMYB* in *Beta vulgaris* [39] and *Medicago truncatula* [40] also function as flavonol biosynthesis regulators. The expression of the MYB transcription factor (TF), glutathione S transferase (GST), 4-coumarate-CoA ligase (4CL), and WD repeat genes is higher in red-fleshed apples than in green apples, supporting the observation that the red-fleshed apples accumulate flavonoid and anthocyanin accumulation at comparatively higher levels than green apples [41]. A transcription factor like c-MYB that regulates the biosynthesis of anthocyanins is encoded by the C1 gene in maize, the first MYB gene discovered in a plant. The anthocyanins are essential to coloring flower petals and other plant structures, whereas proanthocyanidins are necessary for coloring seed coats [42, 43]. The MYB gene is responsible for controlling the synthesis of anthocyanins and proanthocyanidins, which affects plant structure and seed colour, respectively [44, 45].

## Conclusion

It has been established that members of the MYB gene family play many regulatory roles in controlling how plants respond to diverse biotic and abiotic stresses. Root transcriptome analysis of *Psidium guajava* × *Psidium molle* hybrid generated 2.64 GB data. In silico mining including motifs and expression of guava MYBs was carried out in the present study. A total of 15 MYB family members were identified. They were unevenly distributed among chromosomes in guava probably with the occurrence of gene duplication. The exclusive significant higher expression of the *MYB23* gene (only expressed in root) in wilt-resistant PGPM indicates that it might play a crucial role to confer the wilt resistance in PGPM. Furthermore, the expression patterns of some MYB demonstrated that MYB might participate in the regulation of fruit ripening, seed and root development. Future guava fruit quality will be enhanced with the help of the identification and study of MYB genes. The insights from these findings may help with future functional studies of the MYB genes to clarify their biological functions in guava.

## Supplementary Information

**Additional file 1: Figure S1.** Ancestor chart for GO: 0005634. GO: 0009507. A chlorophyll-containing plastid with thylakoids organized into grana and frets, or stroma thylakoids, and embedded in a stroma. **Figure S2.** Ancestor chart for GO: 0009507. GO: 0012505. A collection of membranous structures involved in transport within the cell. The main components of the endomembrane system are endoplasmic reticulum, Golgi bodies, vesicles, cell membrane and nuclear envelope. Members of the endomembrane system pass materials through each other or through the use of vesicles. **Figure S3.** Ancestor chart for GO: 001250. GO: 0005615.That part of a multicellular organism outside the cells proper, usually taken to be outside the plasma membranes, and occupied by fluid. **Figure S4.** Ancestor chart for GO: 0005615.

## Data Availability

The data generated during the process of this work are available upon request.

## Abbreviations

PG: *Psidium guajava*
PGPM: Hybrid (*Psidium guajava* × *Psidium molle*)
MYB: Myeloblastosis
DNA: Deoxyribonucleic acid
CDS: Coding sequence
NCBI: National Center for Biotechnology Information
cDNA: Complementary deoxyribonucleic acid
RT-PCR: Reverse transcription PCR
Taq: *Thermus aquaticus*
TBE: Tris-Borate-EDTA
Trp: Tryptophan
WD: Beta-transducin repeat
