# Supplementary material for: In-silico mining and characterization of MYB family genes in wilt-resistant hybrid guava (Psidium guajava × Psidium molle)
Source: J Genet Eng Biotechnol. 2023 Jun 30;21:74. doi: 10.1186/s43141-023-00528-3 (PMC10313629; doi:10.1186/s43141-023-00528-3)
Supplement: Supplementary file 1 — Additional file 1: Figure S1. Ancestor chart for GO: 0005634. GO: 0009507. A chlorophyll-containing plastid with thylakoids organized into grana and frets, or stroma thylakoids, and embedded in a stroma. Figure S2. Ancestor chart for GO: 0009507. GO: 0012505. A collection of membranous structures involved in transport within the cell. The main components of the endomembrane system are endoplasmic reticulum, Golgi bodies, vesicles, cell membrane and nuclear envelope. Members of the endomembrane system pass materials through each other or through the use of vesicles. Figure S3. Ancestor chart for GO: 001250. GO: 0005615.That part of a multicellular organism outside the cells proper, usually taken to be outside the plasma membranes, and occupied by fluid. Figure S4. Ancestor chart for GO: 0005615. [file 43141_2023_528_MOESM1_ESM.docx]

**GO: 0005634**

A membrane-bounded organelle of eukaryotic cells in which chromosomes are housed and replicated. In most cells, the nucleus contains all of the cell's chromosomes except the organellar chromosomes, and is the site of RNA synthesis and processing. In some species, or in specialized cell types, RNA metabolism or DNA replication may be absent.

**Fig S1** Ancestor chart for GO: 0005634

**GO: 0009507**

A chlorophyll-containing plastid with thylakoids organized into grana and frets, or stroma thylakoids, and embedded in a stroma.

**Fig S2** Ancestor chart for GO: 0009507

**GO: 0012505**

A collection of membranous structures involved in transport within the cell. The main components of the endomembrane system are endoplasmic reticulum, Golgi bodies, vesicles, cell membrane and nuclear envelope. Members of the endomembrane system pass materials through each other or though the use of vesicles.

**Fig S3** Ancestor chart for GO: 001250

**GO: 0005615**

That part of a multicellular organism outside the cells proper, usually taken to be outside the plasma membranes, and occupied by fluid.

**Fig S4** Ancestor chart for GO: 0005615
